# Insufficient Acetyl-CoA Pool Restricts the Phototrophic Production of Organic Acids in Model Cyanobacteria

**DOI:** 10.3390/ijms252111769

**Published:** 2024-11-01

**Authors:** Dawei You, Faiz Rasul, Tao Wang, Maurycy Daroch

**Affiliations:** School of Environment and Energy, Peking University Shenzhen Graduate School, Shenzhen 518055, China; dw.you@stu.pku.edu.cn (D.Y.); frasul@pku.edu.cn (F.R.); wangtaojosh@stu.pku.edu.cn (T.W.)

**Keywords:** cyanobacteria, 3-hydroxypropionate, 3-hydroxybutyrate, succinate, fumarate, malate, TCA cycle, acetyl-CoA, bioeconomy, *Synechococcus*

## Abstract

Cyanobacteria are promising biological chassis to produce biochemicals such as carboxylic acids and their derivatives from CO_2_. In this manuscript, we reflected on cyanobacterial acetyl-CoA pool and TCA cycle as an important source of precursor molecules for the biosynthesis of carboxylic acids such as 3-hydroxypropionate, 3-hydroxybutyrate, succinate, malate, fumarate and free fatty acids, each of which is an important platform chemical for bioeconomy. We further highlighted specific features of the cyanobacterial TCA cycle, how it differs in structure and function from widely described TCA cycles of heterotrophic model organisms, and methods to make it more suitable for the production of carboxylic acids from CO_2_. Currently, the yields of these compounds are significantly lower than those in heterotrophic organisms and it was concluded that the primary cause of this can be attributed to the limited flux toward acetyl-CoA. Strategies like overexpressing pyruvate dehydrogenase complex or introducing synthetic bypasses are being explored to overcome these limitations. While significant progress has been made, further research is needed to enhance the metabolic efficiency of cyanobacteria, making them viable for the large-scale, sustainable production of carboxylic acids and their derivatives.

## 1. Sustainable Production of Carboxylic Acids and Their Hydroxy Derivatives Using Cyanobacteria

The production of chemical building blocks using microorganisms has long been considered an important step toward a more sustainable bioeconomy [1]. Typical feedstocks to produce these compounds are carbohydrates derived from food crops and, to a lesser extent, waste streams such as glycerol or hydrolysates from lignocellulosic residues. On the other hand, the latter are problematic in processing and typically result in lower yields [2,3]. Moreover, the application of food crops as primary carbon feedstocks comes with additional environmental and moral issues, resulting in undesirable changes in land use and elevated food prices.

In contrast, cyanobacteria are photosynthetic autotrophic bacteria, which can yield energy and essential building blocks directly from sunlight and atmospheric carbon dioxide. Recent advances in genetic engineering have enabled the integration of heterologous enzymes and metabolic pathways for a variety of biofuels and biochemicals in cyanobacteria [4,5]. To date, the most notable examples of coupling the photosynthetic carbon fixation to the production of biochemicals are isoprene [6], ethanol [7], 2,3-butanediol [8], and sucrose [9]. Moreover, while the genetic engineering of cyanobacteria significantly lags behind model heterotrophic chassis, entire pathways have been successfully expressed in model unicellular cyanobacteria [10].

Among essential chemical building blocks, carboxylic acids and their hydroxy derivatives play a pivotal role in the transition toward bioeconomy [1]. These compounds can be broadly divided into those that are directly synthesized from acetyl-CoA like 3-hydroxypropionate and 3-hydroxybutyrate and the intermediates of the tricarboxylic acid cycle (TCA or Krebs cycle). Both classes of compounds are important precursor molecules that could be used for the synthesis of various more complex chemicals that have applications as bioplastics, polymers, solvents, adhesives, coatings, food additives, pharmaceuticals, and many more [11]. The application of these platform chemicals in various industries highlights their importance in promoting sustainable solutions, reducing reliance on fossil fuels, and contributing to a circular economy. Meanwhile, fatty acids, essential building blocks of all cells, are considered as both important platform chemicals and compounds of high potential as nutraceuticals [12]. Depending on their chain length they could be classified as short-chain (up to 6 carbon atoms), medium-chain (between 6 and 10 carbon atoms) and long-chain fatty acids (over 12 carbon units). These molecules can be further integrated in an array of secondary metabolites with potentially unique properties [13].

To effectively produce these organic acids in cyanobacteria, it is important to understand the biosynthesis of the precursor molecules in these phototrophic organisms, the peculiarities of their acetyl-CoA-derived metabolism, and the cellular import and export characteristics of cyanobacteria.

## 2. Acetyl-CoA Pool in Cyanobacteria

Acetyl-CoA is one of the major central metabolic precursors alongside glyceraldehyde-3 phosphate and pyruvate. The acetyl group is linked to coenzyme A through a thioester linkage, the hydrolysis of which releases a substantial amount of energy to fuel cellular metabolism [14]. The molecule provides two carbon units for the biosynthesis of many compounds, including fatty acids and amino acids, and is the gateway to the TCA cycle. Intracellular acetyl-CoA pool is generated via the oxidative decarboxylation of pyruvate, a process facilitated by the tightly regulated pyruvate dehydrogenase complex (PDHc), or through the anaerobic pyruvate ferredoxin oxidoreductase (PFOR) [15] or pyruvate formate lyase pathway (PFL) [16]. The pyruvate formate lyase pathway is more commonly found in anaerobic or facultatively anaerobic bacteria, where it plays a role in fermentative metabolism, and is not typically found in cyanobacteria.

*Escherichia coli* is the most utilized organism for the biosynthesis of biochemicals and biofuels derived from acetyl-CoA [17,18,19]. In heterotrophic organisms, such as *E. coli*, acetyl-CoA is the main metabolite formed from glycolytic carbon flow through pyruvate into PDH, and supplemented with building blocks from the breakdown of amino acids and fats. Meanwhile, cyanobacteria exclusively rely on the stream of de novo-synthesized photosynthetates through the central metabolic pathway. Since the energy needs of an organism are fulfilled by photosystems, the reaction catalyzed by PDHc is tightly controlled [20]. One of the limitations of the PDH complex is its strict regulation at both transcriptional and translational levels. Moreover, it is also influenced by NADH/NAD^+^ redox ratios and substrate level allosteric inhibitions, which can significantly limit the carbon flux to the acetyl-tight regulation of the complex, negatively impacting the carbon flux toward the TCA cycle and all the products that are derived from the intermediates of the cycle. This is probably because the reaction results in carbon losses and the generation of reducing equivalents that, under photoautotrophic conditions, are not essential.

## 3. TCA Cycle in Cyanobacteria Differs from That in Heterotrophs

The tricarboxylic acid cycle is a key metabolic route consisting of a series of catabolic reactions that generate reducing equivalents for ATP formation and important precursor molecules for the biosynthesis of cellular components. The cycle is also embedded into a broader metabolic network, making the TCA cycle essential for other metabolic processes, including nitrogen metabolism.

In aerobic bacteria (including cyanobacteria), the TCA cycle has two main roles: (a) generating NADH through the oxidation of carbon derived from acetyl-CoA, which then supplies electrons to oxidative phosphorylation; and (b) the formation of intermediates such as oxaloacetate, 2-oxoglutarate (2-OG) or succinate, all of which are necessary for the generation of cellular building blocks such as amino acids [21,22].

The TCA cycle is initiated through a citrate synthase-catalyzed reaction of acetyl-CoA and oxaloacetate (OAA). It subsequently goes through a series of reactions that release two carbon atoms as CO_2_, and concludes with regeneration of the OAA [23]. The canonical TCA cycle has eight steps catalysed by citrate synthase, aconitase, isocitrate dehydrogenase, 2-oxoglutarate dehydrogenase complex, succinyl CoA ligase, succinate dehydrogenase, fumarase, and malate dehydrogenase [24]. Interestingly, studies have shown that cyanobacteria have an intact but unconventional TCA cycle [25]. In this alternative variant, the absence of 2-oxoglutarate dehydrogenase (2-OGDH) and succinyl-CoA synthetase is compensated with other reactions. Two alternative enzymes, 2-oxoglutarate decarboxylase (2-OGDC, OgdA) and succinate semialdehyde dehydrogenase (SSADH, SsaD), catalyze the transformation of 2-oxoglutarate (2-OG) into succinate. These two reactions, present in nearly all cyanobacteria, corrected the long-held misconception that these organisms have an incomplete TCA cycle. It also provided important insights into the physiological significance of the TCA cycle in these microbes [25,26,27].

The absence of 2-OGDH in cyanobacterial metabolism has significant effects, leading to the emergence of several bypass pathways that compensate for this missing enzyme. These alternative routes involve the metabolism of succinic semialdehyde, γ-aminobutyric acid (GABA) or glyoxylate [28,29,30]. For instance, in *Synechococcus* sp. PCC 7002 (now reclassified as *Picosynechococcus* sp. PCC 7002), succinic semialdehyde dehydrogenase and 2-oxoglutarate decarboxylase are expressed in place of 2-oxoglutarate dehydrogenase and succinyl-CoA ligase, respectively [26]. In contrast, *Synechocystis* sp. PCC 6803 compensates for the absence of these enzymes by utilizing a GABA shunt [27,31]. Another important finding has been in model strain *Synechocystis* PCC 6803, where analysis of the enzymatic kinetics of the two putative enzymes completing the TCA cycle revealed that the majority of the flux in the TCA cycle of this cyanobacterium is channeled through malic enzyme into pyruvate rather than through malate dehydrogenase into oxaloacetate [32]. Nevertheless, another variant of the TCA cycle in cyanobacteria, found in strains such as *Chlorogloeopsis fritschii* PCC 9212, relies on the glyoxylate shunt [33]. Interestingly, it has been observed that enzymes like fumarase, succinate dehydrogenase, and malic enzyme are dispensable under both dark and light conditions [30].

While diazotrophic cyanobacteria can fix atmospheric N_2_, many other species rely on its assimilation from ammonium, nitrite, nitrate, urea, arginine and glutamine, and the TCA cycle is fundamentally connected to this nitrogen metabolism through the production of essential molecules from its intermediates oxaloacetate and 2-oxoglutarate. In non-diazotrophic cyanobacteria, ammonium is assimilated via the glutamine synthetase cycle, where 2-oxoglutarate serves as the final nitrogen acceptor [34].

The metabolism of nitrogen and the TCA cycle in cyanobacteria are thus connected and largely reliant on the availability of the former. Consequently, under nitrogen-starved conditions, cyanobacteria cannot produce sufficient proteins for growth and instead prioritize glycogen synthesis as carbon storage. In such a situation, the activity of the TCA cycle, which normally generates precursor metabolites, is reduced. Instead, more carbon flux is directed upstream toward the biosynthesis of ADP-glucose, which is subsequently used for glycogen polymerization [35]. Another role of the TCA cycle is the regeneration of fumarate, but in some cyanobacteria such as *S. elongatus* PCC 7942, this function of the pathway is dispensable, resulting in its secretion into the growth medium [36]. Meanwhile, in *Synechocystis* sp. PCC 6803, it has been observed that the flow of metabolites [29,30] via 2-oxoglutarate to succinate is lower than in a regular TCA cycle, as it is rerouted through the GABA shunt [24,29,30]. The flux balance analyses have shown that under photoautotrophic conditions, only a marginal flux occurs through these bypass pathways [37].

In conclusion, cyanobacterial metabolism shows a strong connection between the carbon-fixing Calvin–Benson–Bassham (CBB) cycle and the TCA cycle. Since the CBB cycle plays the primary role in carbon delivery and photosystems are essential for energy generation, the TCA cycle is less critical for maintaining cellular energy balance in cyanobacteria than it is in heterotrophic organisms. On one hand, this reduced reliance on the TCA cycle suggests that cyanobacteria, at least when grown autotrophically, could tolerate more extensive genetic modifications in this pathway compared to heterotrophs [21]. On the other hand, the naturally lower flux toward the TCA cycle could have a negative impact on the productivity of compounds derived from the molecules downstream of pyruvate when cells are grown autotrophically.

## 4. Photosynthetic Production of Short Acetyl-CoA-Derived Organic Acids

In phototrophically grown cyanobacteria, the biosynthesis of organic acids occurs through either reductive or oxidative processes. Short-chain organic acids, such as 3-hydroxypropionate (3HP) and 3-hydroxybutyrate (3HB), are synthesized directly from acetyl-CoA. These reactions require an input of energy in the form of NAD(P)H and can be enhanced with ATP, allowing for the conservation of photosynthetically fixed carbon (see Table 1).

Acetate is the simplest organic acid that is an exception from this rule. The molecule can be synthesized from acetyl-CoA using three different mechanisms. Many cyanobacteria possess two of these pathways: acetyl-CoA synthetase, and a combination of phosphotransacetylase and acetate kinase [38]. This metabolic toolkit enables both the uptake of acetate during mixotrophic growth [39,40] and its secretion [41,42]. Since both pathways generate ATP, they typically do not operate under photosynthetic conditions; instead, they are common during dark self-fermentation. Studies on dark self-fermentation and mixotrophy are ubiquitous, yet beyond the scope of this manuscript that focuses exclusively on the photosynthetic production of organic acids. Interestingly, to date, only a single study in which a glycogen-deficient strain of *Picosynechococcus* sp. PCC 7002 was found to secrete acetate under photoautotrophic conditions [43]. To the best of our knowledge, no further targeted engineering efforts have been performed to maximize acetate production under photoautotrophic conditions. There are, however, considerable metabolic engineering efforts focusing on longer and more functional organic acids, which are presented below.

### 4.1. 3-Hydroxypropionate

3-hydroxypropionate (3HP), a C3 hydroxycarboxylic acid, is an important platform chemical in bioeconomy. 3HP was selected as one of the top chemical building blocks derived from biomass by the Department of Energy (DOE) [1]. It also attracted significant interest from the scientific community and industry alike. 3HP is a versatile compound with numerous synthetic applications. Many essential chemicals, including acrylic acid, 1,3-propanediol, acrylamide and methyl acrylates, can be derived from 3HP. These compounds are key building blocks for the manufacturing of many important products such as fibers, contact lenses, diapers, fabric coatings, super absorbent polymers and biodegradable plastics.

Current microbial production methods rely on heterotrophic organisms such as *Escherichia coli* and *Klebsiella pneumoniae*. These organisms primarily utilize glucose and glycerol as fermentation substrates [44]. Since these carbon sources are originally produced from food crops, such as corn or rapeseed, it is desirable to investigate even more sustainable carbon alternatives and develop the technologies of their direct production from CO_2_ and sunlight using phototrophic microorganisms.

**Figure 1:**
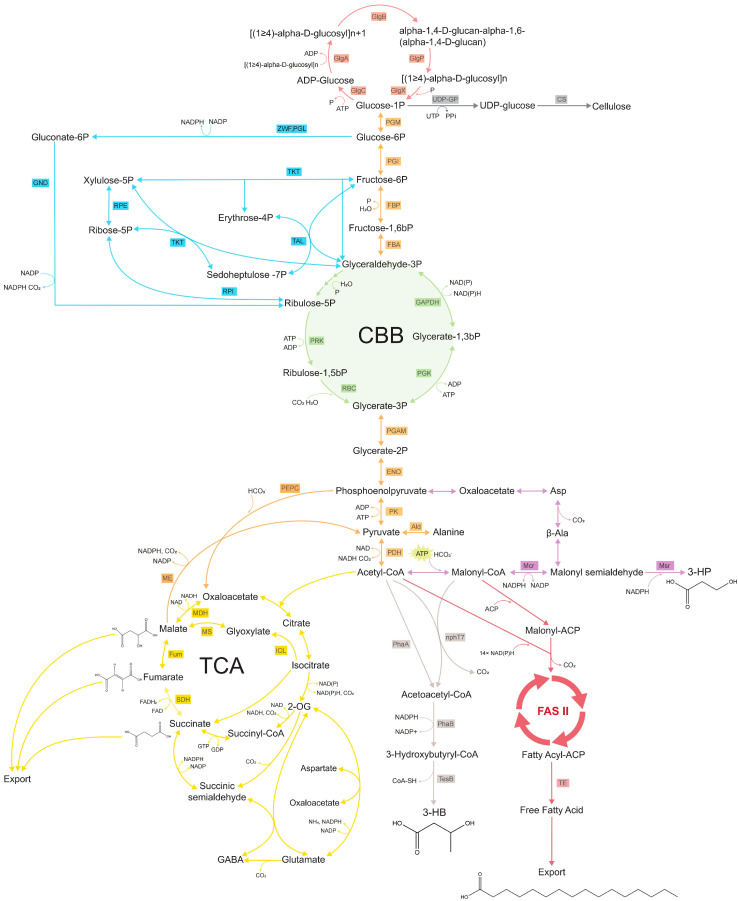
Cyanobacterial carbon metabolism pathways for carboxylic acid production. Tricarboxylic acid cycle and its components are marked in gold, 3-hydroxypropionate in purple, 3 hydroxybutyrate synthesis pathways in grey, Calvin–Benson–Bassham cycle in green, oxidative pentose phosphate in blue, non-CBB glycolysis pathway in orange and glycogen metabolism in red.

In principle, 3HP in cyanobacterial chassis could be synthesized using three alternative approaches (Figure 1). The three biosynthetic routes include the glycerol, malonyl-CoA and β-alanine pathways. To date, only two latter pathways have been successfully explored for the heterologous synthesis of 3HP in cyanobacteria. The limited use of the glycerol pathway in cyanobacteria can be attributed to the oxygen sensitivity of its key enzyme, glycerol dehydratase. The most common approach to synthesize 3HP in cyanobacterial chassis is using the malonyl-CoA pathway. The flow of acetyl-CoA is initially directed into malonyl-CoA, subsequently into malonyl-CoA semialdehyde and ultimately into 3-hydroxypropionate. These biochemical reactions are performed using two enzymes, Malonyl-CoA reductase (*Mcr*) and Malonate semialdehyde reductase (*Msr*). The two enzymes are not found in native cyanobacteria and need to be expressed heterologously. These genes are typically present in thermophilic chemolithoautotrophic archaea such as *Metalospheraea sedula* or *Sulfolobus tokodaii* or in anoxygenic thermophilic photosynthetic bacteria, e.g., *Chloroflexus aurantiacus*. In each of these organisms, they constitute essential sections of their respective carbon fixation cycles. The heterologous expression of Mcr-Msr that enables 3HP production from CO_2_ has been successfully shown in two unicellular model cyanobacteria: *Synechococcus* PCC 7942 and *Synechocystis* PCC 6803 (Table 2).

In *Synechococcus* PCC 7942, multiple combinations of *Msr* and *Mcr* were tested to determine the optimal selection of genes to produce 3HP. Among all the combinations tested, the DC3 strain comprising *M. sedula* Msr and *S. tokodaii* Mcr, expressed under the control of a single IPTG-inducible P_Trc_ promoter, resulted in the highest observed 3HP titer of 659 mg/L after 16 days of cultivation, albeit at a considerable growth penalty [45]. Even higher titers were possible through the engineering of another model strain: *Synechocystis* PCC 6803. The organism was modified by introducing a gene of *Mcr* derived from *C. aurantiacus* under the control of a strong P_cpc560_ promoter combined with the overexpression of a native NAD(P)^+^ transhydrogenase (pntAB) that provides a pool of optimal reductant to achieve a 3HP production of 751.86 mg/L. This construct was further improved with the strengthening of the flux toward malonyl-CoA thanks to the overexpression of Acetyl-CoA carboxylase composed of AccB, AccC, AccA and AccD supplemented with the biotinilase-encoding gene (*birA*). This combination of genes yielded the highest productivity reported to date of 837 mg/L. Interestingly, the deletions of potentially competitive pathways toward PHB and acetate biosynthesis yielded only marginal gains and were not statistically significant improvements.

When it comes to the alternative approach for 3HP production, with the β-alanine pathway, the most productive strain of this type, the DC1 variant of *Synechococcus* PCC 7942, could reach a titer of 186 mg/L after 16 days of cultivation without exhibiting growth penalty [45]. Interestingly, introducing this modification into malonyl-CoA-dependent strain DC3, resulting in strain EL63, yielded no further improvement in the titer of 3HP. This implies that the activities of enzymes comprising the heterologous pathway were not a limiting factor in 3HP production in cyanobacteria. The likely causes of low productivity could be attributed to product toxicity, limitations in precursor molecule availability or other factors.

Despite employing, and sometimes integrating, these two approaches, typical titers of 3HP obtained in cyanobacteria do not exceed 1 g per liter. These productivities fall significantly short of those in heterotrophic model organisms where productivities are typically in tens of grams per day [67]. This can be primarily attributed to a relatively low acetyl-CoA pool in autotrophic organisms. An additional factor could be the lack of temperature alignment between the optimal growth temperature of mesophilic cyanobacterial chassis and enzymes derived from thermophilic bacteria and archaea.

### 4.2. 3-Hydroxybutyrate

Longer hydroxy acids branching directly from acetyl-CoA have also been synthesized in the cyanobacterial chassis. 3-hydroxybutyrate (3HB), a C4 hydroxycarboxylic acid, is another important platform chemical. It primarily serves as a monomer for the synthesis of biodegradable bioplastics used in an array of packaging, medical, agricultural, and textile industries. Currently, 4-hydroxybutyrate is primarily produced from the hydrolysis of microbially produced polyhydroxybutyrate using acids or enzymes [68,69]. The process, despite its efficiency, first requires the microbial production of the biopolymer, often using heterotrophic strains and feedstocks derived from food crops. This is followed by the multiphase hydrolysis of polyhydroxyalkanoate granules and purification of the hydrolysate to yield monomers that are later repolymerized into a more tailored bioplastic. A simplified process that directly converts CO_2_ into monomeric building blocks using energy derived from sunlight is, therefore, desired.

This C4 carboxylic acid, one carbon longer than 3HP, is synthesized from acetoacetyl-CoA (Figure 1), a result of a condensation of two acetyl-CoA molecules. Its biochemical pathway is composed of thiolase (phaA) and acetoacetyl-CoA reductase (phaB) genes typically found in the operon *phaCAB* responsible for the biosynthesis of polyhydroxyalkanoates. This operon is present in many PHB-producing cyanobacteria, including *Synechocystis* PCC 6803.

Early works on *Synechocystis* PCC 6803 have shown that whilst the simple expression of an *E. coli* thioesterase results in a titer of 20.6 mg/L during photosynthetic production [47], few relatively straightforward genetic modifications can significantly improve the productivity of the strain. The deletion of endogenous PHB synthases phaC and phaE, combined with the amplification of the native flux toward PHB through the overexpression of phaA and phaB under the control of a relatively strong P_tac_ promoter, was effective in yielding 533.4 mg/L of 3HB after 21 days of cultivation. In a follow-up study, acetoacetyl-CoA reductase (phaB) was identified as the primary bottleneck restricting productivity. Addressing this bottleneck through optimization of the gene’s ribosome binding site and making it complementary to the 3′-terminal sequence of *Synechocystis* PCC 6803 16S rRNA resulted in a (R)-3HB titer of 1.84 g L^−1^ within 10 days and peak productivity of 263 mg L^−1^ day^−1^ [49].

In another model unicellular strain, *Synechococcus* PCC 7942, the baseline titer of 120 mg/L of 3HB in 5 days of cultivation was achieved through the expression of the essential *phaA*, *phaB* and *Thl* genes [48]. Major improvements in this productivity were possible through the introduction of the ATP-driving force module that replaced *phaA* with *nphT7*, a gene encoding acetoacetyl-CoA synthase. *NphT7*, together with native acetyl-CoA carboxylase, drives the irreversible synthesis of acetoacetyl-CoA. An additional driving force boosted the productivity two-fold but resulted in growth inhibition, which could be alleviated in the subsequent iteration by increasing thioesterase expression. The final and most successful strain capable of synthesizing 1.2 g/L 3HB contained both the driving force module resulting from the activity of nphT7 and phaA from the original construct that allows for the better control of the flux.

Overall, the productivities of cyanobacteria optimized for the biosynthesis of 3HB exceed 1 g L^−1^ and are higher than those of 3HP, but still fall short when compared to heterotrophic organisms such as *E. coli* or *S. cerevisiae*, where the productivities typically exceed a gram per hour [70]. There may be many reasons why phototrophs offer lower productivities of 3HB, most notably lower cellular densities in the bioreactor scale and a lower pool of acetyl CoA.

## 5. Photosynthetic Production of Organic Acids Derived from the TCA Cycle

In contrast to the production of organic acids from acetyl-CoA, which typically consumes cellular resources like ATP and NAD(P)H while retaining fixed carbon, organic acids derived from the TCA cycle, such as fumarate, malate, and succinate, are synthesized via the oxidative forward branch of the cycle under photosynthetic conditions. This oxidative process generates NADH and releases previously photosynthetically fixed carbon. This differs from the production of the same compounds through dark self-fermentation where the reductive branch of the TCA cycle is used, NADH is consumed and carbon is conserved. Since this manuscript is centered around the photosynthetic production of organic acids, subsequent discussion will focus on the oxidative branch of the pathway.

### 5.1. Fumarate and Malate

Fumarate is a C4 hydroxycarboxylic diacid and an important platform chemical selected among the top 12 value-added chemicals from biomass. Traditionally, it has been chemically synthesized from maleic acid and its precursor maleic anhydride [71]. It is primarily used as an acidity regulator in the food industry but also in detergents, animal feed, and pharmaceuticals. Because of the need to transition into greener production, a direct process of its biosynthesis from CO_2_ would be desired, but reports on direct fumarate production using cyanobacteria are scarce.

Fumarate, an essential component of the TCA cycle (Figure 1), is produced during anabolism. Recently, a growth-coupled strategy was developed that allowed for the photosynthetic production of 1 mM titer (116 mg L^−1^) of fumarate thanks to the deletion of the *fumC* gene of *Synechocystis* PCC 6803 [50]. The gene is responsible for converting fumarate into malate in the TCA cycle. This productivity was later slightly improved by deleting the *zwf* gene encoding glucose-6-phosphate dehydrogenase in the oxidative pentose phosphate pathway. The protein encoded by the gene converts glucose-6-phosphate into 6-phosphoglucono-δ-lactone. A productivity boost associated with the deletion of the *zwf* gene was observed primarily at night and was attributed to the redirection of cellular energy reserves into the TCA cycle, when light harvesting does not provide ATP.

Another TCA cycle-derived C4 hydroxycarboxylic diacid from the DOE Top 12 value-added chemicals from biomass is malate (Figure 1) [1]. The molecule is used in the food, pharmaceutical, and agriculture industries, and, like other C4 diacids, is an important chemical precursor typically produced chemically from maleic anhydride. An interesting approach to the photosynthetic production of malate was proposed by Hu et al. [51]. In this work, fast-growing *Synechococcus* UTEX 2973 was engineered by introducing phosphoenolpyruvate carboxykinase (*pck*) and malate dehydrogenase (*mdh*) genes to generate a shunt that provides additional ATP and carbon fixation capacity during the photosynthetic production of malate [51]. In the first iteration of this solution, engineered strain *SH006* could produce approximately 200 μM (27 mg L^−1^) malate after 9 days of cultivation, which was further increased to 260 μM (35 mg L^−1^) in strain *SH008* after introducing a stronger promoter for phosphoenolpyruvate carboxykinase.

Elsewhere, in a follow-up study to the one on fumarate [50], a growth-coupled strategy was developed for the biosynthesis of 1.2 mM (161 mg L^−1^) of malate in a double-deletion mutant of *Synechocystis* PCC 6803 lacking malic enzyme (me) and malate dehydrogenase (mdh) [52]. A near-complete degree of the conversion of fumarate into malate was achieved with the expression of fumarase gene *fumC* originating from *E. coli* to boost the native flux toward malate.

### 5.2. Succinate

Succinate is the final of three dicarboxylic acids from the DOE Top 12 chemicals from biomass that is a TCA cycle-derived intermediate (Figure 1) [1]. It is an essential platform chemical that can be converted into major industrial compounds 1,4-butanediol, butadiene, gamma-butyrolactone and tetrahydrofuran. Just like other diacids, malate and fumarate, it is primarily produced chemically from maleic anhydride, but its fermentative production using *Actinobacillus succinogenes*, *Escherichia coli* or *Corynebacterium glutamicum* is also mature. Applications of succinate are vast, spanning food, cosmetics, pharmaceutical and biopolymer applications.

The photosynthetic production of succinate in *Synechococcus* PCC 7942 has been achieved by expressing genes encoding α-ketoglutarate decarboxylase (*kgd*) and succinate semialdehyde dehydrogenase (*gabD*) in S. elongatus PCC 7942 under the control of P_trc_ promoter. The biosynthesis of 120 mg/L of succinate was achieved with a significant growth penalty, most likely due to α-ketoglutarate depletion [54]. This shortage of the essential precursor has been alleviated with the expression of phosphoenolpyruvate carboxylase (ppc) and citrate synthase (gltA) from *Corynebacterium glutamicum* in said cyanobacterial strain. The strategy was successful, and the final producer strain, LAN3, was capable of biosynthesizing 430 mg/L of succinate in 8 days without a significant growth penalty. In a parallel study, another group utilized the CRISPR-Cas9 system for the simultaneous knockout of glucose-1-phosphate adenylyl transferase (*glgC*) responsible for glycogen synthesis and replacing it with *ppc*-*gltA* cassette [58] analogous to the one described above [54]. The engineered strain cultivated under nitrogen deprivation was capable of synthesizing 0.435 mg/L of succinate, a value significantly lower than the one based on an alternative approach proposed by another group [54]. The exact reasons for this discrepancy are unknown. Another CRISPR-based study utilized CRISPRi to downregulate the genes *glgc*, *sdhA* and *sdhB* responsible for glycogen and fumarate biosynthesis in *S. elongatus* PCC 7942, respectively. The most successful strategies employed in this work were based on the suppression of both succinate dehydrogenases *sdhA* and *sdhB*, and resulted in titers between 0.580 and 0.630 mg/L [72], similar to other studies employing CRISPR-Cas technology.

In the final paper from these studies [58], a similar approach resulted in drastically different outcomes. The best-producing strain from this line of research, LAN3 [54], was subjected to a series of CRISPRi experiments targeting the key competing pathways of succinate production [53]. In line with the previous study, the repression of *sdhA* and *sdhB* resulted in the highest gains of succinate yield, exceeding 30% in each case and reaching a succinate titer of 576 mg/L in the case of the latter. In the next step, the compound effect of *sdhB* and *glgC* suppression was studied. The resulting strain, CR8, was capable of synthesizing 632 mg/L of fumarate in 8 days without any growth penalties. When scaling up the cultivation of CR8 from flask to photobioreactor, the succinate titer reached 4.8 g/L after 28 days when air was supplemented with 5% CO_2_, a major improvement that also indicates the need for testing multiple cultivation and carbon delivery approaches. Interestingly after a month of cultivation, cells became non-viable due to unknown reasons. The culture medium itself could be reinoculated with new cells to continue succinate production for a final of four cycles and a titer of 8.9 g/L, representing, by far, the highest succinate titer reported to date.

When it comes to another model cyanobacterium, *Synechocystis* PCC 6803, the approaches were similar. In early works, a series of *Sdh*-deletion mutants were prepared to increase the succinate productivity of this model strain [56]. One of the deletions targeting the *sll1625* gene resulted in a maximal titer of 420 mg L^−1^ succinate. The deletion mutant grown in high CO_2_ accumulated both succinate and glycogen and, unlike the wild-type under the same cultivation conditions, did not undergo bleaching. In the follow-up study, different cultivation conditions of the same were tested [59]. A detailed study suggests that light–dark cycles promote succinate titers and that no pH control during the photosynthetic production of succinate has a positive impact on the production of the metabolite. Elsewhere, the introduction of glyoxylate to *Synechocystis* PCC 6803 shunt two genes, *aceA* and *aceB*, encoding isocitrate lyase and malate synthase, respectively [55]. The engineered strain, constructed in the background of phosphoenolpyruvate-overexpression strain 2P constructed earlier [73], resulted in the strain capable of synthesizing 140 µg mL OD under 20 µE m^−2^ s^−1^ in a nitrogen-depleted medium. This is two-fold higher than the same cells grown in a nitrogen-rich medium, but also only a third of the dark fermentation in a nitrogen-replete medium. Even better results were obtained when nitrogen limitation was replaced by an addition of succinate dehydrogenase inhibitor, 2-thenoyltrifluoroacetone (TTA). Production exceeding 400 µg mL^−1^ OD^−1^ was obtained in this setting and further increased over three-fold by transitioning to mixotrophic growth using acetate [73]. Similar combinations were even more successful when transitioning from photosynthetic growth into dark and dark anoxic self-fermentation, but these studies are beyond the scope of the manuscript.

With the continuous development of new cyanobacteria for the production of biochemicals, attention has shifted toward the application of fast-growing chassis. One of these organisms is *Synechococcus elongatus* PCC 11801, recently proposed as a promising salt-tolerant and rapidly growing phototroph [74]. A very respectable yield of 0.6 g L^−1^ in 5 days has been achieved through a previously suggested combination of the overexpression of α-ketoglutarate decarboxylase, succinate semialdehyde dehydrogenase and phosphoenolpyruvate carboxylase [57], resulting in the strain SA3. Based on this construct, further improvements were made. The combined overexpression of seven genes (*OgdA*, *SsaD*, *PEPC*, *gltA*, *SBPase*, *YjjP* and *YjjB*) and the deletion of *glgA* and *sdhB* allowed for a succinate titer of 0.93 g L^−1^ in variant SA9. The resultant strain was also characterized by similar growth parameters to the wild-type and low fumarate titers.

## 6. Photosynthetic Production of Free Fatty Acids

Fatty acids are long-chain carboxylic acids synthesized through the condensation of acetyl- and malonyl-CoA precursors. The process involves the repetitive addition of two-carbon units derived from malonyl-CoA, which itself is formed from acetyl-CoA. This mechanism of synthesis requires considerable investment of ATP and NAD(P)H, and makes fatty acids one of the most chemically reduced hydrocarbons formed through biological pathways. As a result, fatty acid synthesis has been the subject of extensive basic and applied research. In the early 2000s, the concept of microalgal biodiesel, based on the transesterification of fatty acids stored in the triglycerides of oleaginous microalgae, gained considerable attention [75]. However, the promise of large-scale sustainable biodiesel production from microalgae ultimately fell short of the high initial expectations.

Unlike oleaginous microalgae, cyanobacteria do not synthesize considerable amounts of triglycerides and alternative strategies, based on genetic engineering, have been developed for the export and harvesting of long-chain fatty acids. An originally innovative and now ubiquitous approach involves engineering cyanobacteria to secrete free fatty acids (FFAs), which can later serve as valuable bioproducts or biofuel precursors. The interest in FFA secretion originates from earlier work on *E. coli*, where disrupting fatty acid β-oxidation led to their extracellular secretion [60]. However, due to the fundamental differences in carbon metabolism and the incomplete β-oxidation pathway in cyanobacteria [63], a different strategy was needed. In cyanobacteria, fatty acid recycling is governed by acyl–acyl carrier protein synthases (Aas) [60]. Therefore, deleting these proteins, alongside overexpressing thioesterases, has become a successful strategy to increase FFA production and secretion in these microorganisms [60,63].

Early works demonstrated an effective synthesis of 197 mg of FFAs per liter of growth medium by an extensively engineered strain of *Synechocystis* PCC 6803 (variant SD277) [60]. The strain, featuring a combination of heterologous thioesterases and the deletion of endogenous carbon sinks, is, to this date, one of the best producers of free fatty acids reported in the literature. Initial attempts to achieve similar productivities in another model strain, *Synechococcus elongatus* PCC 7942, were less successful. The expression of a truncated *E. coli* in a strain, denoted as SE02, yielded an organism capable of achieving titers exceeding 70 mg of free fatty acids per gram of dry biomass [61]. In the follow-up study, attempts were made to increase the flux toward fatty acids. The two putative rate-limiting steps in channeling the carbon flow toward fatty acid synthesis, the overexpression of Rubisco and acetyl-CoA carboxylase, were tested. Although volumetric productivity did not improve, carbon partitioning (measured as FFA mass versudry cell weight) was superior to the parental strain [62]. These findings suggest that exploring various thioesterases and reducing carbon flow into competing sinks, like polyhydroxyalkanoates and cyanophycin, may be more effective for FFA production than inducing metabolic flux toward fatty acid synthesis.

Further studies applied this strategy to other photosynthetic organisms. A fast-growing marine strain, *Synechococcus* PCC 7002 (*Picosynechococcus* sp. PCC 7002), was engineered with a knockout of *fadD*, combined with the expression of truncated *E. coli* thioesterase (*‘tesA*) and Rubisco (*rbcLS*) under the control of *S. elongatus* PCC 7942 *psbAI* promoter. The resultant strain was capable of synthesizing 131 mg/L of FFAs [63]. Elsewhere, the same strain was used as a producer of medium-chain free fatty acids by exchanging *E.coli* thioesterase with one with an altered substrate specificity. The resultant strain could produce 4.4 mg L^−1^ day^−1^ of lauric acid (C12) and a total fatty acid titer of approximately 95 mg L^−1^ [64]. Interestingly, attempts to further increase carbon partitioning through the deletion of ADP–glucose pyrophosphorylase responsible for glycogen synthesis were only marginally successful. This reinforces previous finding that indicates that the deletion of downstream metabolic carbon sinks is more useful in increasing the flux toward fatty acids than altering glycogen metabolism that is placed upstream of carbon-fixation reactions.

Initial works have shown that the titers of secreted free fatty acids in *Synechocccus elongatus* PCC 7942 lagged behind other cyanobacteria and resulted in growth impairment, but the exact causes were unclear [65]. More detailed studies revealed excessive intracellular free fatty acid accumulation as a leading cause of this phenomenon. An approach that helped alleviate the issue was the deletion of the *wzt* gene, responsible for the synthesis of hydrophilic O-antigen on the cell surface. This genetic manipulation increased free fatty acid productivity to 2.7 mg L^−1^ h^−1^. Despite the productivity improvement, the engineered strain showed growth retardation and ultimately short-lived phenotype due to the instability of PSII, indicating the need for alternative approaches to free fatty acid secretion [65]. One approach, tested in *Synechocystis* PCC 6803, involved targeting thioesterases to the inner membrane using a membrane scaffolding system based on *Synechocystis* Lgt protein. This significantly increased FFA production to 331 mg/L, nearly three times that of a strain expressing free thioesterase (121 mg/L) [66], highlighting the importance of cellular localization for free fatty acid synthesis. Recent strategies for improving FFA secretion in transgenic cyanobacteria include integrating lipase A to hydrolyze membrane lipids. This approach, tested in *Synechocystis* PCC 6803, resulted in 24.5% carbon partitioning as FFAs per dry cell weight [76], confirming its potential to boost FFA production.

## 7. Outlook

Photosynthetic production of carboxylic acids in cyanobacteria has been an active area of research in the last decade. The overview of the current state of the field reveals that the volumetric productivities are very low compared to the heterotrophic counterparts. There may be numerous ways how this situation could be addressed, and multiple approaches can be taken to improve it. Here, we will reflect on the current selection of producer strains and discuss the potential solutions to low yields.

Two model organisms, *Synechocystis* PCC 6803 and *Synechococcus* PCC 7942, still dominate as primary production hosts for organic acids, most likely due to more mature genetic engineering methods. Interestingly, in recent years, alternative organisms such as fast-growing *Synechococcus* UTEX 2973 and *Synechococcus* PCC 11801, were also used.

When it comes to overall titers achieved during photosynthetic production, they are typically within a range of high mg to low g per liter of medium. Among all the organic acids, the highest yields have been achieved in cases of 3HB (1840 mg/L), fumarate (2700 mg/L) and succinate (8900 mg/L). These three compounds have been produced in organisms that already have most of the desired pathway components in place and typically require rechanneling the carbon flux toward a higher conversion efficiency. Therefore, looking into the future selecting among native strains that already have essential components of the pathway in place may be a promising strategy.

The photobiosynthesis and secretion of free fatty acids from cyanobacteria have been an active field of work for over a decade, and despite numerous attempts to engineer stable and efficient producers of these molecules, similar issues persist. The overall productivity remains in low hundreds of mg per liter of growth medium; albeit due to their hydrophobic character, their purification is greatly simplified. Moreover, most approaches that reduce the intracellular content of fatty acids and promote their secretion result in unstable phenotypes that are mostly applicable to short production cycles. Better control of the secretion process with inducible promoters, riboswitches of dedicated circuits could be a way forward in obtaining higher yields of free fatty acids from these phototrophic bacteria.

Generally, the yield per unit volume achieved with cyanobacteria is much lower, sometimes by three orders of magnitude, than that achieved by heterotrophs. For example, the biotechnological production of succinate using heterotrophic organisms is within several grams per liter per hour [77], whereas the typical per-hour productivity of photosynthetically grown cyanobacteria is between 3 and 6 mg of succinate in most recent works [53]. These comparisons, while showing one side of the gap to fill before photoautotrophic chassis can match the current performance of model heterotrophic chassis, neglect important differences between the two systems: cell densities. Heterotrophic cells can be grown in high cellular densities that cannot be achieved by photoautotrophs due to their need for a constant supply of light. Whilst heterotrophic cultures can typically exceed 50 g L^−1^ in batch cultivation [78], actively growing cyanobacterial cultures rarely approach 10 g L^−1^ [79]. In this context, per cell comparison between the phototrophic chassis and model heterotrophs becomes an order of magnitude better but still two orders of magnitude lower than those of heterotrophs. The underlying causes must, therefore, be different.

An abundant precursor pool is essential for the high-level production of desired chemicals. Analysis of genome-scale models reveals that the acetyl-CoA level in *Synechocystis* PCC 6803 is lower than that in *Escherichia coli*. The potential reason may be differences in the overall structure of the metabolic network. For example, acetyl-CoA is used in 22 reactions in *Escherichia coli* and only 14 reactions in *Synechocystis* PCC 6803. These differences may explain the restriction of acetyl-CoA levels in *Synechocystis* PCC 6803 and its lower quantity than in *Escherichia coli* [80].

We, therefore, speculate that the major factor that prevents the high-yield production of organic acids and their derivatives from CO_2_ using cyanobacterial chassis is the low flux toward the TCA cycle. When a biosynthesis pathway in cyanobacteria is introduced or overexpressed, titers of chemicals derived from pyruvate, e.g., 5.5 g L^−1^ ethanol [7], are much higher than those derived from acetyl-CoA, e.g., 100 mg L^−1^ fatty alcohols [81], because the intracellular content of acetyl-CoA is less than 5% of the abundant pyruvate content [5].

Acetyl-CoA in cyanobacteria is primarily produced from pyruvate by a pyruvate dehydrogenase complex [82]. The low acetyl-CoA content may, therefore, result from the potential low catalytic efficiency of PDHc or a weak driving force due to the generally low activity of the TCA cycle in cyanobacteria under photoautotrophic conditions [26]. Alternatively, the acetyl-CoA control network differs in autotrophs and heterotrophs to limit the excessive transfer of carbon into an intermediate that has a secondary role in central metabolism [82]. Consequently, several earlier studies applied dark-fermentation nutrient-deficient conditions to enhance the intracellular acetyl-CoA availability via activating glycogen degradation [83]. Alternatively, introducing co-substrates that directly feed into the acetyl-CoA pool has also been tested as a promising strategy to increase the product yield [84]. Despite the potential of these approaches to increase the yield, these cultivation strategies do not allow for continuous growth and stable productivity.

Thus, enriching the flux to the acetyl-CoA pool appears to be a key step to enhance the production of downstream products [80] without restricting the process to anaerobic cultivation conditions [82].

Therefore, overcoming the regulation and increasing flux from pyruvate to acetyl-CoA has been a strategy to enlarge the acetyl-CoA pool. A study on *Synechococcus elongatus* PCC 7942 showed that by overexpressing the native PDHc, total PDHc activity was increased two-fold and intracellular acetyl-CoA content reached 2.6-fold that in the control strain. When biosynthesis pathways for two acetyl-CoA-derived products, acetate and isopropanol, were introduced into the acetyl-CoA-enhanced strain separately, this resulted in a 7-fold and a 3.8-fold improvement in product titer, respectively. In addition, the overexpression of different *PDH* genes in *S. elongatus* PCC 7942 was tested but resulted in no improvement in PDH activities. The introduced genes originated from *Escherichia coli* (*aceEF*, *lpd*), *Synechocystis* PCC 6803 (*pdhABCD*) [85].

Intriguingly, eukaryotic yeast has evolved to solve the tight regulations through the existence of organelles such as mitochondrion and peroxisome [86]. Thus, the metabolic engineering of a key node between pyruvate and acetyl-CoA mimicking such compartmentalization is desired for the construction of efficient bio-solar cell factories that require the high-level acetyl-CoA as a precursor [87]. To further explore this concept, a synthetic acetate-acetyl-CoA/malonyl-CoA (AAM) bypass to overcome a rate-limiting step of PDHc and increase the acetyl-CoA pools to acetone has been introduced [88]. The AAM-bypass was designed to combine a synthetic acetoacetyl-CoA (AA)-bypass that generates acetyl-CoA and NADPH from pyruvate and an artificial PPS/PPC/MMC-CO_2_-fixing module composed of phosphoenolpyruvate synthetase (PPS), phosphoenolpyruvate carboxylase (PPC) and methyl malonyl-CoA carboxyltransferase (MMC). The module fixes CO_2_ through PPC and rearranges the carbons of pyruvate and malonyl-CoA into oxaloacetate and acetyl-CoA using MMC [88]. By integrating a pyruvate decarboxylase, an aldehyde dehydrogenase and an acetylation-tolerant acetyl-CoA synthase into the wild-type strain, acetyl-CoA content increased 6.5-fold by day 8 of cultivation and, interestingly, the pyruvate content was also 2-fold higher than the wild-type strain [88].

## 8. Conclusions

The photosynthetic production of carboxylic acids in cyanobacteria holds promise, and titers exceeding 1 g L^−1^ are currently achievable for some of these products. These values, however, are still below the productivity characteristic of heterotrophic microorganisms. Whilst significant improvements have been made in recent years and numerous approaches have been tested, the major issue that prevent the high-yield production of carboxylic acids and their derivatives from CO_2_ appears to be the low metabolic flux toward acetyl-CoA, which limits the yield of acetyl-CoA-derived products. Hopefully, in the future, existing strategies involving overexpressing the pyruvate dehydrogenase complex (PDHc) or introducing synthetic bypasses to overcome the tight limitation of the flux will be widely applied, and new strategies enabling the replenishing of the acetyl-CoA pool with inorganic carbon will be developed.

## Figures and Tables

**Table 1:** Resources needed for the biosynthesis of organic acids derived from acetyl-CoA and TCA cycle intermediates.

Chemical	Pathway	Enzymes Involved	Resources Needed from Acetyl CoA
3HP (C3)	Malonyl-CoA pathway	Acetyl-CoA carboxylase (*Acc*), Malonyl-CoA reductase (*Mcr*), Malonate semialdehyde reductase (*Msr*)	HCO_3_^−^, 1 ATP, 2 NADPH
3HP (C3)	β-alanine pathway	Phosphoenolpyruvate carboxylase (*PepC*), aspartate aminotransferase (*AspC*), PLP-independent aspartate decarboxylase (*PanD*), PLP-dependent aspartate decarboxylase (*Adc*), β-alanine aminotransferase (*SkPYS4*), Malonate semialdehyde reductase (*Msr*)	HCO_3_^−^, 1 ATP, 2 NAD(P)H
3HB (C4)	3-hydroxybutyrate (3HB) pathway	Thiolase (*PhaA*), Acetoacetyl-CoA reductase (*PhaB*)	1 Acetyl-CoA, 1 NADPH
3HB (C4)	ATP driving module for 3HB	Acetoacetyl-CoA synthase (*NphT7*), Acetyl-CoA carboxylase (*Acc*)	1 Acetyl-CoA, 1 ATP, 1 NADPH
Succinate (C4)	Oxidative branch of TCA cycle	Citrate synthase (G*ltA*), Aconitase (*AcnA*), Isocitrate dehydrogenase (*Icd*), 2-oxoglutarate decarboxylase (*OgdA*) and succinate semialdehyde dehydrogenase (*SsaD*)	Oxidative branch reaction, NADH and CO_2_ formation
Succinate (C4)	Oxidative branch of TCA cycle	Oxidative branch of TCA cycle supplemented blocked with succinate dehydrogenase deletion (∆*sdh*)	Oxidative branch reaction, NADH and CO_2_ formation
Succinate (C4)	Oxidative branch of TCA cycle + *kgd*, *gabD*	Oxidative branch of TCA cycle supplemented with 2-ketoglutarate decarboxylase (*Kgd*), succinate semialdehyde dehydrogenase (*GabD*)	Oxidative branch reaction, NADH and CO_2_ formation
Succinate (C4)	Oxidative branch of TCA cycle + *aceA*, *aceB*	Oxidative branch of TCA cycle supplemented with isocitrate lyase (*AceA*); malate synthase (*AceB*)	Oxidative branch reaction, NADH and CO_2_ formation
Free Fatty Acids (palmitate) (C16)	Fatty acid synthesis II + *tesA*	Acetyl-CoA carboxylase (*Acc*), Malonyl-CoA transacylase (*Mcat*) 3-Ketoacyl-ACP synthase (*Kas*), 3-Ketoacyl-ACP reductase (*FabG*), 3-Hydroxyacyl-ACP dehydrase (*FabZ*, *FabA*), Enoyl-ACP reductase (*FabI FabK*), Acyl carrier protein (ACP), acyl- ACP thioesterase (*tesA*)	7 Acetyl-CoA, 7 ATP, 14 NAD(P)H

**Table 2:** Summary of the photosynthetic production of carboxylic acids in cyanobacteria.

Chemical	Producer Strain	Genotype	Titer	Reference
3HP	*Synechococcus elongatus* PCC 7942_DC3	P_Trc_: *msr* (*M. sed*)*, mcr* (*S. tok*) in NSI *(Synpcc7942_2498*)	659 mg L^−1^	[45]
3HP	*Synechococcus elongatus* PCC 7942_DC1	P_LlacO1_: *Ae_adc, Skpyd4, msr* (*M. sed*)*, ppc, aspC* in NSII (*Synpcc7942_0084*)	186 mg L^−1^	[45]
3HP	*Synechococcus elongatus* PCC 7942_EL63	P_Trc_: *msr* (*M. sed*)*, mcr* (*S. tok*) in NSI (*Synpcc7942_2498*) P_Trc_: *msr* (*M. sed*)*, mcr* (*S. tok*) in NSI (*Synpcc7942_2498*)	665 mg L^−1^	[45]
3HP	*Synechocystis* sp. PCC 6803_SMPA	P_cpc560_-*mcr-pntAB-*TrbcL in S2 (*slr1704* and *sll1575*) and *accBCAD-birA* through *pJS*	837.18 mg L^−1^	[46]
3HB	*Synechocystis* sp. PCC 6803_TABd	*ΔphaE* (*slr1829*)*, ΔphaC* (*slr1830*)*,* P_tac_*-tesB-kanR* at site S2 (*slr1704* and *sll1575*)*, CmR-*Ptac*-phaA* (*slr1993*)*-phaB* (*slr1994*) at S4 (*slr1992*)	533.4 mg L^−1^	[47]
3HB	*Synechococcus elongatus* PCC 7942_KU21	P_trc_ *nphT7, tesB, phaB, phaA* in NSI (*Synpcc7942_2498*) P_LlacO1_:*pptesB* in NSII (*Synpcc7942_0084*)	1200 mg L^−1^	[48]
3HB	*Synechocystis* sp. PCC 6803_R154	*CmR-*P_tac_*-phaA-*(RBSopt)*-phaB1* at S1 (*slr1495* and *sll1397*)*,* P_psbA12_-P_tac_-*tesB-T1-KanR* at S2 (*slr1362 and sll1274*)*, ΔphaE* (*slr1829*)*, ΔphaC* (*slr1830*)	1840 mg L^−1^	[49]
Fumarate	*Synechocystis* sp. PCC 6803	*ΔfumC* (*slr0018*) and *Δzwf* (*slr1843*)	116 mg L^−1^	[50]
Malate	*S. elongatus* UTEX 2973_FH389	*∆nblA::AsPck (L), ∆TP70::AsMdh (L), EmR, CmR;* pEM-CF53 *: pEM,* T5*-pck,* T5*-mdh,* T5*-prk,* T5*-CA (H), T5-RbcL, pEM-CF53,*	34.86 mg L^−1^	[51]
Malate	*Synechocystis* sp. PCC 6803_WD199	*Δmdh* (*sll0891*)*, Δme* (*slr0721*)*, OE fumC* (*JW1603*)	160.91 mg L^−1^	[52]
Succinate	*Synechococcus elongatus* PCC 7942_CR8	P_trc_:*:gabD, kgd, gltA, ppc; SpecR* in NSI (*Synpcc7942_2498*) P_LlacO1_:*:dCas9;* P_trc_*::*sgRNA*(glgC-1);* P_trc_*::* sgRNA*(sdhB-2); KanR* in NSII (*Synpcc7942_0084*)	8900 mg L^−1^	[53]
Succinate	*Synechococcus elongatus* PCC 7942_LAN3	Ptrc*::gabD* (*JW2636*)*, kgd* (*SYNPCC7002_A2770*)*, gltA, ppc;* in NSI (*Synpcc7942_2498*)	430 mg L^−1^	[54]
Succinate	*Synechocystis* sp. PCC 6803_ 2P_C	*Δ* *psbA2::pepC-KmR pepC-pepC slr1068::CmR*	1616 ± 183 mg L^−1^	[55]
Succinate	*Synechocystis* sp. PCC 6803_∆sll1625	*∆sll1625*	420 mg L^−1^	[56]
Succinate	*Synechococcus elongatus* PCC 11801_SA9	*△DOP62_03525:: SmR* P_psbaI_ *OgdA* (*Synpcc7002_A2770*) T_rbcl_ P_psbaIII_ *SsaD* (P25526) T_rrnb_ P_cpcb300_ *pepc* (*Synpcc7942_2252*) T_rbcl_*, ΔDOP62_03790::*P_rbcl400_ *gltA* (*Synpcc7942_0612*) T_rbcl_ P_rbcl400_ *SBPase* (*Synpcc7942_0505*) T_rbcl_ *KanR, ΔDOP62_11515::GmR* P_prbcl300M_ T_rbcl_ *yjjP*(*POADD2*) P_cpcb200_ *yjjB* (*POADD5*) T_lac_	930 mg L^−1^	[57]
Succinate	*Synechococcus elongatus* PCC 7942-Δglgc::ppc::gltA 0XN	*Δglgc* (*Synpcc7942_0603*)*::ppc* (*Synpcc7942_2252*)*::gltA* (*Synpcc7942_0612*) *0XN*	0.435 ± 0.035 mg L^−1^	[58]
Succinate	*Synechocystis* sp. PCC 6803_∆sll1625	*∆sll1625*	472 mg L^−1^	[59]
Succinate	*Synechococcus elongatus* PCC 7942	*∆sdhA* (*sll1625 and slr1233*)*, ∆sdhB* (*sll0823*)	0.58–0.63 mg L^−1^	[56]
Succinate	*Synechococcus elongatus* PCC 11801_SL3338	NSI*::*P_cpcB_*-efe-*T_rrnB_	1044.18 µmole g_DCW_ ^−1^ h^−1^ *	[46]
Free Fatty Acids	*Synechocystis* sp. PCC 6803_SD277	*slr1609::*PpsbA2 *‘tesAΔ*(*slr1993-slr1994*)*::*Pcpc*-accBC* Prbc accDA Δsll1951::*PpsbA2 *Uc fatB1* Prbc *Ch fatB2Δ*(*slr2001-slr2002*)::*PpsbA2 *ChfatB2Δslr1710::*PpsbA2* *Cc fatB1Δslr2132::*Ptrc *tesA137*	197 mg L^−1^	[60]
Free Fatty Acids	*Synechococcus elongatus* PCC 7942	*ΔSynpcc7942_0918::E. coli* (*‘tesA*)	75 mg g_DCW_^−1^ *	[61]
Free Fatty Acids	*Synechococcus elongatus* PCC 7942	*ΔSynpcc7942_0918::E. coli* (*‘tesA*)*;* Ptrc*-fat1* (*XM_001696567*)*-rbcLS (Synpcc7942_1426, Synpcc7942_1427*)*,* PLlacO1*-accBCDA* (*Cre17.g715250.t1; Cre08.g359350.t1; Cre12.g519100.t1; Cre12.g484000.t1*)	25 mg L^−1^	[62]
Free Fatty Acids	*Picosynechococcus elongatus* PCC 7002	*ΔfadD* (*SYNPCC7002_A0675*)*;* Ptrc*-‘tesA-*PpsbAI*-rbcLS (Synpcc7942_1426, Synpcc7942_1427*)	131 mg L^−1^	[63]
Free Fatty Acids	*Picosynechococcus elongatus* PCC 7002	*ΔglgC::aphII, ΔfadD::*PcpcBA*-fatB1-aadA*	95 mg L^−1^	[64]
Free Fatty Acids	*Synechococcus elongatus* PCC 7942_dAS1T∆wzt	*∆aas::*PnirA*-‘tesA ∆wzt*	100 mg L^−1^	[65]
Free Fatty Acids	*Synechocystis* sp. PCC 6803_mACT	P_mAcT_ *pBS-NSU0168-*P_cpcB_*-ssSecLacLgt-AcTesA-P_cpcB_-Km-NSD0168*	331 mg L^−1^	[66]

* can not be converted into mg/L using data provided in the article.

## Data Availability

The original contributions presented in the study are included in the article; further inquiries can be directed to the corresponding author.
